#  ‘I’m doing it for myself’: Using a smartphone-based exercise service during the COVID-19 lockdown in the Faculty of Health Sciences, University of the Witwatersrand, South Africa

**DOI:** 10.17159/2078-516X/2021/v33i1a9053

**Published:** 2021-04-30

**Authors:** G Torres, N Neophytou, P Fourie, X Buntting, D Constantinou, PJ-L Gradidge

**Affiliations:** Centre for Exercise Science and Sports Medicine, Faculty of Health Sciences, University of the Witwatersrand, Johannesburg, South Africa

**Keywords:** physical activity, home-based exercise, compliance

## Abstract

**Background:**

Sufficient physical activity (PA) lowers poor health outcomes, with data showing these protective effects in populations under varying levels of lockdown during the COVID-19 pandemic. The advent of online PA programmes has created novel opportunities to offset the deleterious effects of inactivity. However, data are limited and the readiness and acceptance of such technology is unknown. These authors nevertheless noted an opportunity to investigate this approach based on promising emerging data at the time of the hard lockdown in South Africa.

**Objective:**

This exploratory study investigated the engagement and perceptions of a smartphone application to promote health and fitness in a sample of employees at a South African university.

**Methods:**

Employed members of staff (n=15) of the University of the Witwatersrand were recruited through email invitation during the hard Level 5 COVID-19 lockdown in 2020. Individualised home-based PA programmes were prescribed through a mobile application for a period of eight weeks. Researchers qualified in Biokinetics provided online supervision of the exercise sessions during the intervention. Participants were asked to complete a self-reported questionnaire about their use of the application. Thematic analysis was used to understand these responses.

**Results:**

Lack of motivation was perceived to have a negative effect on participation in the online PA programme. Only one participant reported using the mobile application consistently during the study period, while half of the participants reported having trouble with the usage of the application. The participants frequently mentioned the need for technical support and further engagement from the clinicians supervising the PA programme to ensure use and progression. Staff identified issues with connectivity and already having too many phone applications (apps) amongst the reasons for the technical difficulties.

**Conclusion:**

This study demonstrates the challenges and potential for the uptake of online PA interventions during COVID-19 and, despite its small sample size, the data provide important lessons learned that will be used as information in further investigations.

The benefits of physical activity (PA) in general, in confinement and during pandemics, has previously been reported and is still relevant during the current wave of the global Coronavirus 2019 (SARS Cov-2) (COVID-19) pandemic.^[1]^ South Africa’s response to the pandemic was rapid and stringent, limiting national economic activity and movement of people. This approach was supported nationally and was particularly important as the health sector prepared for the upswing of the disease’s presence in central ‘hot-spots’, such as city centres and environments with populations living in crowded settings. Sports participation, access to gyms and fitness facilities were prohibited during the lockdown, resulting in limited options for PA and exercise, and therefore concerns regarding rising inactivity levels. Emerging evidence; nevertheless, advocated for continued PA to maintain optimal cardiometabolic and immune health during the pandemic,^[2]^ while other data support obtaining sufficient PA to prevent poor disease outcomes in those individuals contracting COVID-19, compared with that of matched sedentary patients.^[3]^ Home-based exercise for cardiac rehabilitation and maintaining health is now a focused area which was promoted before countries introduced lockdowns.^[4]^

Smartphone PA mobile apps and activity trackers have been used pre-COVID and their applicability during the COVID-19 pandemic is self-evident. Sensors in smartphones and activity trackers enable users to easily access PA programmes and monitor physical activity levels remotely via automated and continuous self-monitoring and feedback. A recent meta-analysis investigated the effect of activity trackers and mobile apps with the features mentioned above in increasing physical activity in healthy adults (18–65 years old).^[5]^ The meta-analysis found that mobile app and tracker users were more active than control groups by an average of 1 850 additional steps per day. The interventions reviewed in the meta-analysis included a variety of smartphone apps and activity trackers, as well as other components of engagement, such as emails, human involvement via phone calls or face-to-face meetings, and text messaging. Furthermore, interventions studied included different behaviour change techniques (e.g. ‘feedback and monitoring;’ ‘rewards and threats’). Importantly, the meta-analysis highlighted that the apps and trackers that were complemented by human personalisation or text-messaging were significantly more effective.

The offer of the smart-phone PA application (in circulation since 2008) was based on the quick implementation of the lockdown and the immediate need for staff members to have an option of exercising during this period. The intention of this study was to offer a mobile app that could be used to access PA home-based programmes and learn this information from the users of the smartphone application.

## Methods

### Participants

The Wits Metro Wellness Gym (WMWG) at the Centre for Exercise Science and Sports Medicine, University of the Witwatersrand (Wits), in the City of Johannesburg, offers sports medicine and exercise science services to employees of the Faculty of Health Sciences and the general community. Current facility members and Faculty staff were offered a PA solution when the facility closed and the COVID-19 lockdown began in South Africa.

### Procedures

Ethical clearance for the study was received from the Wits Human Research Ethics Committee (Medical) (certificate M200843). The targeted population was invited via email to download the mobile application. The application offered a daily ‘Workout of the Day (for Home)’ of easy, medium, or high intensity levels.

The mobile application was connected to a Cloud platform that allowed Biokineticist staff to assign, record and track PA. Participants could also connect a third-party device (e.g. wearable fitness devices) to the application and record all activity/exercise.

Biokinetics staff could connect with the members weekly via the ‘coach’ section on the mobile application that facilitates messaging between the health professional and participant. The biokineticist was able to track PA levels on the online platform and send ‘nudges’ and ‘cues’ to participants. This could only be done with participants who engaged with and downloaded the mobile application. Therefore, not all participants were ‘coached’ during this study intervention.

### Questionnaire

A questionnaire link was sent to participants for completion and to provide data to understand the use of the technology. Self-reported PA levels were calculated using a physical activity index (PAI). The PAI is a non-exercise equation^[6]^ consisting of three components: frequency, amount, and intensity of PA. The product of the variables categorises PA as (i) low, (ii) moderate or (iii) high.

### Statistical analysis

Descriptive statistics were conducted for the quantitative portion of the analysis and reported as mean (+SD), or median (interquartile range (IQR)) for skewed data. Statistica version 13.5 (Tibco, USA) was used to perform the quantitative analysis. Thematic analysis was conducted manually to determine the themes for the qualitative component. The themes with participant responses were reported verbatim. Two of the researchers (PJG and GT) determined the themes and reached consensus.

## Results

One hundred and fifty-six individuals downloaded the mobile application although not all continued engaging with the app. Only fifteen participants completed the questionnaire, with a median age of 49 (59) years, mean weight (±SD) of 72.4 ± 15.4 kg, and 67% (n=10) were females and 33% (n=5) were males.

A single participant (n=1, 6.7%) reported using the mobile application regularly during the hard lockdown, while 47% (n=7) did not. Twenty percent (n=3) reported the application as being user-friendly while 20% reported difficulty in using the technology. A similar pattern was observed for the remainder of questions, with some participants commenting about receiving inadequate technical support (n=3, 20%). PA activity levels during the first two weeks of lockdown were similar to those before lockdown (n=12, 80%) and the levels during weeks three to five of lockdown were similar to those before and during lockdown (n=11, 73%). PA levels during weeks six to eight of lockdown were similar to those before and during lockdown (n=12, 80%), and finally, PA activity levels increased progressively during the lockdown (n=9, 60%). The same number of participants agreed and disagreed (i.e. 13% for each) that the exercises were updated and they progressed throughout the lockdown.

Only one participant showed data on the cloud-based platform of tracking and recording of workouts via the mobile application. Thus, no analysis was performed on this data.

Through thematic analysis of open-ended questions, the following four themes emerged from the participants’ responses on engagement with the mobile application: motivation, support, usability and technical. The verbatim quotes accompanying the themes can be found in the text in italics. Sixty-nine responses were observed.

### Motivation

Most of the responses were linked to the motivation theme (33/68). Slightly over a third (12/32, 38%) responded to having been motivated to engage in PA through the mobile application.

- *‘New workouts were available daily’*
- *‘Yes. Made a schedule’*
- *‘Home exercises’*
- *‘It helped to establish motivation’*
- *‘As no one else in my family does physical exercise, it was hard to do it on my own, while they watch, but I realised later that I’m doing it for myself, and no one else’*
- *‘It helped to have been able to start walking outside again’*
- *‘Have some home workout equipment’*

In contrast, more participants (21/32, 66%) responded that they were not stimulated to exercise during the lockdown.

- *‘No. No one to train with and enjoy equipment’*
- *‘Lack of motivation and equipment*’
- *‘The cold and lack of motivation’*
- *‘Gym being closed’*
- *‘Missed the engagement with others’*
- *‘Appreciate the initiative, but it’s not for me’*
- *‘I haven’t really had the motivation’*
- *‘Boredom, used to regularly attend gym’*

### Support

Some participants reported that there was insufficient support to assist them with physical activity during the lockdown (4/68).

- *‘Was not given to me’*
- *‘Never used - Prefer free flowing exercises’*
- *‘Not preferable to me’*
- *‘I could not find videos on the app relevant to my gym plan’*

### Usability

Twelve of the responses corresponded to the usability of the mobile application. Fifty percent (6/12) stated that the application was user-friendly.

- *‘The app made it easier for programs to do’*
- *‘Easy to use’*
- *‘It’s good’*

The other half of the responses were aligned with not engaging and with difficulty in using the application (6/12).

- *‘Haven’t been using the app, therefore I cannot offer a response’*
- *‘Needs to be more flexible/user-friendly’*
- *I did not use the app as I struggled to set it up’*

### Technical

A fifth (14/68, 21%) of the responses concerned the technical or preference aspect of the application, suggesting the need for technical support.

- *‘Does not link to Discovery (reward medical health scheme)’*
- *‘Phone/data issues’*
- *‘Not tech savvy’*
- *‘Between insurance companies, banking, email, fitness trackers, social media and other important apps, it is not practical to download yet another one’*
- *‘It takes up space on my phone and I am not interested’*
- *‘Prefer my regular apps’*

Other responses were also considered valuable in understanding participant usage of the application, but were not placed into themes (5/68).

- *‘Other commitments’*
- *‘Injury’*
- *‘To some extent. I had an arthroscopy eight weeks into lockdown and am now recovering’*

## Discussion

This study found that the uptake and engagement with the PA app, even though considered user-friendly, was low, with only 6.7% stating that they used the mobile application. The low adherence to PA smartphone applications has been confirmed. ^[7,8]^ Mollee et al. reported 74 % of health app users stopped using an app within ten times of use, and 26 % of health apps were used only once after being downloaded. ^[8]^

A review by Yanga et al. aimed to synthesise the factors influencing PA mobile application adherence and to identify directions for future research in this area.^[7]^ The evidence indicated that 89 distinct factors influenced PA app adherence. These factors were classified into three categories: Personal factors (user-related), Technology features (app-related) and Contextual factors (environmental-related). Subcategories for Personal factors included Demographic and Socioeconomic factors (e.g. age, gender), Psychological factors (e.g. attitude toward the app-based PA intervention) and Health-related factors (e.g. stress, depression). Others were Predefined goals (e.g. a goal to quit smoking) and Benefits sought (e.g. hedonic motivation). Subcategories for Technology features were User experience (e.g. perceived playfulness) and Functions (e.g. PA tracking). Contextual subcategories were related to the Technical context (e.g. concerns about data privacy) and Social context (e.g. community of users). Studies assessing the causality between factors and PA app adherence are rare.

Understanding the factors that influence PA app adherence provide guidance for app-delivered PA interventions and could explain the results of this study. Table 1 highlights some factors that could explain the low app adherence of this study.

Many of the participants in this study reported decreased levels of PA when compared to the pre-lockdown period. As with the findings in this study, other studies have also shown a decrease in PA in adults because of lockdowns.^[9, 10]^ Ding et al.^[11]^ found that in developed countries, community interest in exercise surged immediately following the lockdown, peaked within the first two weeks, then declined but remained at a higher level than before the lockdown. This indicated that despite challenges to an active lifestyle, the COVID-19 lockdown may have led to increases in population-level interest in PA. It is important to acknowledge though that the interest in exercise may not translate into behavioural change.

Only 20% of the participants in this study found the mobile application beneficial with respect to exercising during lockdown. Technical issues, support and motivation were cited as reasons for not using the technology. One cannot differentiate the underlying reasons for lack of motivation or assign this to the app use or any other reasons. Further, these authors cannot determine whether the opportunity to use the app had any influence on the number of reasons given. The availability of exercise programmes on the mobile application was cited as a reason for those using the technology, suggesting that there was a benefit for some participants. As mentioned. Table 1 highlights some factors that could explain the low adherence and engagement with the PA app used in this study, based on available research.

The researchers of this study are not aware of other studies that have investigated the use of mobile application technology and PA levels during the lockdown phase of the COVID-19 pandemic. This study indicates that technology alone may not be sufficient to influence PA levels during a lockdown, but it appears to have some value. Individuals using such technology appear to need the motivation and support of a health and fitness professional, or a place where exercise can be undertaken to maintain PA levels.

### Lessons learned

The following considerations are possible for such a PA app service to be undertaken based on the factors that influence PA app adherence:

- Making a PA application freely available does not ensure that individuals will engage with the technology.
- A better understanding is needed from the personal, technology and contextual characteristics of the participants with whom the PA app intervention is implemented. It is recommended that an initial (even virtual) meeting with a health and fitness professional is used to gather this information.
- The motivations of and the benefits sought by the participants also need to be understood when implementing a PA app intervention.
- The coaching process needs to be set up as part of the intervention. Personalised health-specific text messaging and a PA target/goal setting should accompany a PA app intervention. The intervention should also involve the coach sending frequent goal setting text message reminders.
- The intervention design needs to consider a team-based approach.
- Attitudes of participants towards the PA app intervention (experiential and instrumental) and towards health and fitness should be assessed prior to offering the intervention.
- There may also be a need for developing a user’s positive attitude towards the PA app and informing them of the benefits they can derive from its use as part of the intervention.
- Factors under technology features are useful for PA app developers or PA intervention designers.

### Limitations

- Tracking on the application is dependent on participants submitting PA exercises performed. Participants may have exercised but did not record sessions.
- Connection to a GPS/heart rate device would give the most accurate, objective recording of activity data. This was not possible to do within the constraints of this study.
- A minimal number of participants engaged with the mobile application and thus most participants were not “coached” or “nudged” for a response to their app. The important effect of PA behaviour modification could thus not be applied or assessed.
- The small number of participants in the study.

## Conclusion

This study demonstrated that reported PA levels decreased during the hard lockdown and offering clients a web-based PA tracking and optimising technology must be supplemented with further coach-client interaction to ensure effective maintenance of PA and exercise at home or during a lockdown. The study’s findings demonstrate that the participants seek further guidance with PA guidelines and support with using the technology, indicating opportunities for evidence-based counsel and supervision by appropriately qualified healthcare professionals. Given that the emerging evidence demonstrates an upsurge of psychological stressors during the COVID-19 pandemic^[12]^, the authors recommend urgent PA rehabilitation and intervention to address mental health concerns in survivors of the disease and populations at risk.

Further research is required to determine the most effective exercise regimens for individuals during lockdowns or for home-based programmes.

## Figures and Tables

**Table 1:** Factors influencing physical activity (PA) app adherence in relation to results of this study

Factors*	Comments applicable to this study’s results

**Personal factors**	
**Demographic and socio-economic**	
Age	Research has indicated that older individuals had higher adherence.
Gender	Males have shown higher app adherence than females.
Income	Having higher income was associated with higher adherence. This study could not account for this factor.
Household type	Living with single parents has been associated with lower adherence. This study did not have access to household type information.
**Psychological factors**	
Positive attitude toward the intervention	A positive attitude increased adherence. Attitude towards to the offer of the intervention was not assessed.
Instrumental attitude	App adherence increases if the person believes that it is smart to use the app and be physically active. This was not assessed in the study.
Experiential attitude	App adherence increases if the person believes the app is fun and the advantages outweigh the disadvantages. This was not assessed in the study.
Attitudes towards health and fitness	This factor may be associated with app adherence but these attitudes were not investigated in the study.
Not liking aspects of the app	The participants of this study did not report not liking any aspects of the app. Thus, this factor was unlikely to be associated with the low adherence to the PA app intervention of this study.
**Health-related factors**	
Self-rating general health	Positively associated with adherence. Not applicable to this study’s results.
Regular app use prior	Positively associated with adherence. Not applicable to this study’s results.
Depressive symptoms	Negatively associated with adherence. Depression levels were not measured in this study. Depressive symptoms though have been associated with lockdown and may have contributed to the low app adherence of this study.
Concern about health	Positively associated with adherence. Not applicable to this study’s results.
Smoker status	Positively associated with adherence. Not applicable to this study’s results.
**Pre-defined goals**	
Goal to quit smoking	Lack of this goal was associated with higher adherence. This study did not investigate participants’ goals.
**Benefits sought**	
Personal benefits	Positive associations have been found with this factor and app adherence. This factor was not assessed in this study.
Hedonic motivations	Positive associations have been found with this factor and app adherence. This study’s questionnaire did not address this factor.
Epistemic benefits	Positive associations have been found with this factor and app adherence. Not applicable to explaining this study’s results.
Social integrative benefits (-)	Positive associations have been found with this factor and app adherence. This factor was mentioned in the participants’ responses.
App-related motives	Positive associations have been found with this factor and app adherence. Not applicable to this study.

**Technology features**	
**User experience**	
Perceived playfulness	The study did not assess this feature.
Effort expectancy	The study did not assess this feature.
Lack of interest (-)	Participants did cite this factor as a reason for not engaging.
Time cost (-)	Participants did cite this factor as a reason for not engaging.
Self-efficacy	The study did not assess this feature.
Ease of use (-)	Some participants did have difficulty using the app.
Accurate consistent data	This is a feature of the app used in the intervention.
Pleasurable workouts	Participants did find the workouts in the app used in the study, beneficial.
Experience	The experience of the app was not interrogated.
**Functions**	
PA tracking	This was a feature of the app used in the intervention.
PA goal/target setting	This was a feature of the app used in the intervention.
Progress monitoring	This was a feature of the app used in the intervention.
Visual/auditory cues	The app used in this intervention had these as part of the technology.
Game-like rewards (-)	Some participants did report the lack of rewards as a reason for not using the app.
Coaching features (-)	Coaching support was positively associated with app adherence. This factor was mentioned by the participants. The PA app used in this intervention had coaching features but they were not used.
Social support	The research was equivocal with regards to this factor.
Personalisation	This feature was available on the app used in the study.
Prompting by the app (-)	This was not available with the app used in the study and may have contributed to the low adherence
Goal-setting text message reminders (-)	The app used in the study had the ability to do this via the coach application, but it was not implemented in the study.
Gamification	Not available on the app used in the study.
Push notifications containing a tailored health message (-)	This was not available with the app used in the study and may have contributed to the low adherence
Customisation of exercise	This feature was available on the app used in the study.

**Contextual factors**	
**Technical context**	
Data privacy	This was not cited as negative factor by the participant of the study.
Technical problems (-)	This was a negative factor reported by some participants.
**Social context**	
Community of users	Not applicable to this study.
Team condition (-)	This factor has been cited as having a causal relationship. Our intervention did not have a team component set-up.

* Factors were taken from Yang et al. ^[7]^

(−); may have contributed to the lack of adherence to the PA app intervention.

## References

[1] Simpson RJ Exercise, immunity and the COVID-19 pandemic Available from: https://www.acsm.org/blog-detail/acsm-blog/2020/03/30/exercise-immunity-covid-19-pandemicCited 19 October 2020

[2] Chowdhury R van Daalen KR Franco OH Cardiometabolic health: Key in reducing adverse COVID-19 outcomes Glob Heart 2020 15 1 58 10.5334/gh.879 32923351 PMC7442174

[3] Chen P Mao L Nassis GP Coronavirus disease (COVID-19): The need to maintain regular physical activity while taking precautions J Sport Health Sci 2020 9 2 103 104 10.1016/j.jshs.2020.02.001] 32099716 PMC7031771

[4] Thomas R Beatty AL Beckie TM Home-based cardiac rehabilitation: A scientific statement from the American Association of Cardiovascular and Pulmonary Rehabilitation, the American Heart Association and the American College of Cardiology Circulation 2019 140 1 e69 e89 10.1161/CIR.0000000000000663 31082266

[5] Laranjo L Ding D Heleno B Do smartphone applications and activity trackers increase physical activity in adults? Systematic review, meta-analysis and metaregression Br J Sports Med 2021 55 8 422 432 https://doi:10.1136/bjsports-2020-102892] 33355160 10.1136/bjsports-2020-102892

[6] Kurtze N Rangul V Hustvedt B-E Reliability and validity of self-reported physical activity in the Nord-Trøndelag Health Study: HUNT 1 Scand J Public Health 2008 36 1 52 61 10.1177/1403494807085373 18426785

[7] Yang X Ma L Zhao X Factors influencing user’s adherence to physical activity applications: A scoping literature review and future directions Int J Med Inform 2020 134 104039 10.1016/j.ijmedinf.2019.104039] 31865054

[8] Mollee JS Middelweerd A te Velde SJ Evaluation of a personalized coaching system for physical activity: User appreciation and adherence PervasiveHealth '17: Proceedings of the 11th EAI International Conference on Pervasive Computing Technologies for Healthcarefc May 2017 315 324 10.1145/3154862.3154933

[9] Ammar A Brach M Trabelsi KHC Effects of COVID-19 home confinement on eating behaviour and physical activity: Results of the ECLB-COVID19 International Online Survey Nutrients 2020 12 6 1583 10.3390/nu12061583 32481594 PMC7352706

[10] Giustino V Parroco AM Gennaro A Physical activity levels and related energy expenditure during COVID-19 quarantine among the Sicilian active population: A cross-sectional online survey study Sustainability 2020 12 11 4356 10.3390/su12114356

[11] Ding D Del Pozo Cruz B Green MA Is the COVID-19 lockdown nudging people to be more active: A big data analysis Br J Sports Med 2020 54 20 1183 1184 10.1136/bjsports-2020-102575 32605932

[12] The intersection of COVID-19 and mental health Lancet Infect Dis 2020 20 11 1217 10.1016/S1473-3099(20)30797-0 33038942 PMC7544473

